# Low Demineralized Caseins to Replace Sodium Caseinate for Application in Whipped Creams

**DOI:** 10.3390/foods13233897

**Published:** 2024-12-03

**Authors:** Khadija Florence Dabo, Christine Chèné, Sylvain Prevost, Anne-Laure Fameau, Romdhane Karoui

**Affiliations:** 1 Adrianor, 62217 Tilloy-Lès-Mofflaines, France; f.dabo@adrianor.com (K.F.D.); c.chene@adrianor.com (C.C.); 2 University of Artois, University of Lille, University of Littoral Côte d’Opale, University of Picardie Jules Verne, University of Liège, INRAE, JUNIA, UMR-T 1158, BioEcoAgro, F-62300 Lens, France; 3 Institut Laue-Langevin, 71 Avenue des Martyrs, CS 20156, Cedex 9, 38042 Grenoble, France; prevost@ill.fr; 4 CNRS, INRAE, Centrale Lille, UMET, University of Lille, F-59000 Lille, France; anne-laure.fameau@inrae.fr

**Keywords:** casein, demineralization, structure, foam, emulsification, whipping creams

## Abstract

Caseinate is commonly used in the dairy industry for its stabilizing properties. Its functionalities are largely due to its manufacturing process involving a high level of demineralization that induces casein precipitation. To address this, the researchers are looking for alternatives to respond to consumer demands for high-quality ingredients and increase efficiency. In the present study, we explored low demineralization (0–43%) that preserves the casein integrity to produce caseins that can substitute caseinate in whipping creams (WC). The physicochemical, structural, and functional properties of these caseins were evaluated to assess their viability as surface-active agents in blend-fat whipping cream. The results showed that a demineralization level over 43% significantly impacts the size, secondary structures (β-sheets, β-turns, and α-helices particularly), and surface hydrophobicity that favorably impact the emulsifying properties but diminish the foam stability. WC made with caseins demineralized at 0 and 13% provided stable foam, with the lowest emulsion stability at 0% of demineralization. Using demineralized caseins at 13% offers a sustainable alternative to caseinate in food products requiring stable foams as WCs without significantly altering other desired functionalities such as overrun and emulsion stability. Further research studies into optimizing the demineralization process and exploring applications in other food matrices are suggested.

## 1. Introduction

Milk proteins are widely used in dairy products to play various roles as emulsifiers, stabilizers, and foaming agents, depending on the application. This versatility of these proteins is due to their ability to undergo structural and conformational changes that influence their functional properties [1]. Parameters linked to the environment of the proteins, like pH, temperature, and ionic strength, among others, can also influence their structure [2]. pH has been demonstrated to differentially affect the size, charge, structure, and consequently the functionalities of caseins, depending on their range [3,4].

Milk proteins are composed of two types: caseins and whey proteins. The caseins are the most representative protein in milk (80%) and are divided into four different types (α_S1_-, α_S2_-, β- and κ-caseins, with a weight ratio in cow milk of approximately 4:1:4:1, respectively) that coexist within the micelle with colloidal calcium phosphate [5]. At the natural pH of milk (~6.8), caseins are organized as micelle maintained by hydrophobic interactions and electrosteric stabilization, and pH variation can disturb this equilibrium [5,6]. The demineralization process affects this equilibrium by involving a decrease in milk pH value, which disrupts the phospho-calcic bonds that link casein submicelles together to form micelles. This disruption alters the structure and stability of casein micelles, impacting their functionality in food applications [7]. Subsequently, the pH is neutralized by dialysis or using an alkali, as acidification reduces casein solubility in water dispersions [8]. The pH is adjusted to a range of 6 to 7 to more closely mimic the natural pH of milk, ensuring optimal conditions for maintaining the functional properties of milk proteins. The demineralization process can lead to structural and physicochemical changes in the protein that affect its functionalities, including emulsification, foaming, and gelling, among others [9]. During the acidification step, chemical acids such as citrate, lactate, and gluconate are used [10]. Acidification is carried out within a pH range of 7 to 5, above the isoelectric point fixed at pH 4.6, to prevent casein coagulation and maintain its structural integrity [11]. Nevertheless, the pH can decline to a value below 4.6 to reach a high demineralization rate, as is required for caseinate production. Dialysis is typically employed to restore the mineral equilibrium of milk [9,11,12,13,14]. An alkali can also be used to restore the pH, e.g., in caseinate production. The designation of the caseinate is dependent upon the alkali utilized in its production. For instance, sodium caseinate is the result of the use of sodium hydroxide as an alkali.

pH acidification followed by dialysis revealed that a high level of demineralization significantly reduced the particle size of casein [11,15], altered the secondary structure of caseins [16], and modified protein conformation [11] can affect their functionalities. As previously outlined by Silva et al. [9], demineralization level does not significantly affect the foaming capacity of foams prepared using the double syringe method from casein solutions. However, the stability of the resulting foams is negatively correlated to casein demineralization rate [9]. The research conducted by Lazzaro et al. also demonstrated that an increase in demineralization rate enhances casein emulsifying capacity [14]. However, this adjustment leads to a corresponding decline in the emulsion stabilization capacity, particularly affecting the prevention of creaming, flocculation, and resistance to coalescence [14]. These findings indicate that while highly demineralized caseins demonstrate favorable emulsifying properties, they are less effective as foam stabilizers.

In dairy products, sodium caseinate is the most used protein for its stabilization properties and resistance to heat treatment. Caseinate is obtained from a process of total demineralization of caseins, which confers excellent functionalities [17]. They can be found in several food systems, such as sauces and whipping creams, where they play different roles [18]. In whipping creams, caseinate is incorporated essentially for its emulsion stabilization abilities. Whipping cream is a dairy-based emulsion, which is commonly used for dessert preparations such as Chantilly, ganache, etc. Typically, whipping cream contains 30–40% fat and around 0.5–2% of dairy proteins [19]. After whipping, their volume roughly doubled due to the very well-known process of partial coalescence of fat droplets [20,21]. This trend is primarily due to the high level of saturated fats (~70% of total fat) in the cream but also to the presence of dairy caseins [22]. The findings of Li et al. [23] have demonstrated that caseinate is an effective emulsion stabilizer, preventing coalescence and thereby influencing the foaming properties of whipped creams, which are low [23]. This result is consistent with previous research on the emulsifying and foaming properties of caseinate in dairy emulsions [9,14].

Despite their widespread use in the production of dairy products, caseinates have certain disadvantages. These include their low foam stabilization but also their high chemical manufacture [9,24]. As a result, the food industry is actively seeking alternative ingredients that can overcome these challenges while still meeting the functional demands of food products. This shift is further fueled by growing consumer interest in sustainable and minimally processed foods. A promising alternative is the use of less demineralized caseins, which undergo fewer chemical modifications than caseinates that maintain casein structural integrity and can positively improve both their emulsifying and foaming properties.

This study aims to produce caseins with varying degrees of demineralization (ranging from 0 to 43%) and to compare their properties with caseinates for application in whipping cream.

## 2. Materials and Methods

### 2.1. Casein Fraction and Sample Preparation

Four industrially caseins produced by following the caseinate process (acidification then neutralization using an alkali) with varying demineralization levels (0–13–22–43%), and a commercially available fully demineralized caseinate (used as a reference) were provided by Inleit Ingredients (Curtis, Spain). All protein solutions were prepared with a fixed amount of protein poured into deionized water to attain a final concentration of 10 mg/mL. The samples were first prepared under magnetic stirring (300 rpm) for 12 h at room temperature. Fat-based whipping cream blends were developed at pilot plant scale using 7 wt.% of milk fat, 28 wt.% of palm kernel oil, 1 wt.% of casein powder, and 64 wt.% of skimmed milk, emulsifiers, and stabilizers. The manufacturing process and detailed recipe of the pilot-scale whipping creams were kept confidential as corporate property. Analytical reagent-grade chemicals from VWR were used for all experiments.

### 2.2. Physicochemical Characterization of Caseins

#### 2.2.1. Turbidity Measurements

Turbidity measurements were carried out with a UV/vis spectrometer (Shimadzu, Kyoto, Japan). 10 mg/mL of casein was reconstituted in deionized water under magnetic stirring for a whole night at room temperature. Then, before analysis, the samples were centrifuged at 10,000× *g*^−1^ using SW14R centrifuge (Froilabo, Paris, France) for 10 min at 20 °C and finally filtered (pore size 0.45 µm from Avantor, Leicestershire, UK). Transmission of the samples was determined at a wavelength of 600 nm [25]. A cuvette with a 1 cm pathway was used. All the measurements were conducted at ambient temperature.

#### 2.2.2. Caseins Particle Size

The particle size (Z-average diameter) of demineralized caseins was measured using a Zetasizer Nano-ZS (Malvern Instruments Ltd., Worcestershire, UK). The casein solutions were prepared under identical conditions to those described in Section 2.2.1. Equilibration time before measurements started was set to 2 min in order to ensure the accuracy of the data, and then the size of caseins was measured at room temperature.

#### 2.2.3. Small Angle Neutron Scattering (SANS)

SANS patterns were recorded at D22 beamline at the Institut Laue–Langevin—The European Neutron Source (ILL, Grenoble, France), with a wavelength λ of 0.60 nm (relative full width at half maximum 10%) and two ^3^He multi-PSD tube detectors, one at 1.4 m sample-to-detector distance and the second at 17.6 m, covering a total *q*-range of 0.023 to 6.4 nm^−1^, where *q* is the magnitude of the wavevector (*q* = 4πsin(θ/2)/λ, with *θ* the scattering angle). For this experiment, the samples were prepared in D_2_O to increase contrast and reduce background. Solutions were kept in quartz cuvettes of 1 mm pathway (type 110-QS, Hellma GmbH, Müllheim, Germany) placed on a sample-changer thermalized at 20 °C. Data reduction was performed with the program GRASP V.10.17i [26] to correct for flat field, parallax, transmission, scattering by the empty cell, detector background as measured with an absorber (sintered ^10^B_4_C) at the sample position, using the monitor to normalize data, and with a measure of the direct beam attenuated with a calibrated chopper leading to corrected data in absolute units (cm^−1^), which were radially averaged and binned according to resolution. Data are available at DOI: 10.5291/ILL-DATA.9-10-1846. Contributions from solvent and incoherent scattering were then subtracted.

#### 2.2.4. Mid-Infrared (MIR) Measurements

2 mL of casein solution (1 wt.%) was poured into the ATR cell. MIR spectra were recorded at ambient temperature between 3000 and 900 cm^−1^ at a resolution of 16 cm^−1^ Fourier IR-Tracer 100 (Shimadzu, Duisburg, Germany). The ATR cell used as a sampling accessory has a reflection horizontal crystal made of zinc selenide (ZnSe) with an incidence angle of 45° and a number of reflections of 10. To improve the signal-to-noise ratio, 32 scans were accumulated for each spectrum. The background spectrum was scanned at the beginning of measurement by pouring the ATR cell with Millipore Q-purified water and using the same instrumental conditions as that used during spectra acquisition. The same procedure was used for scanning blank spectra. The secondary structures of the five caseins were calculated on data obtained. The FTIR data was corrected by derivatization (second derivative of the absorbance in the Amide I region between 1700 and 1600 cm^−1^). Within the Amide I band, several regions were analyzed, including side chain (1614–1600 cm^−1^), β-sheet (1637–1614 cm^−1^), random coil (1645–1637 cm^−1^), α-helix (1664–1645 cm^−1^); β-turn (1681–1664 cm^−1^); aggregated β-sheet (1700–1681 cm^−1^). The area of each component representing secondary protein structures was estimated using LabSolutions software version 2.02 (June 2013).

#### 2.2.5. Fluorescence Measurements

The surface hydrophobicity of caseins was determined using 8-anilino-1-napthalenesulfonic acid ammonium salt (ANS) as a fluorescence probe. 10 mg/mL of casein was reconstituted in deionized water under magnetic stirring at ambient temperature for a night. A volume of 2 mL of the casein solution was pipetted into a quartz cell. Subsequently, the cell was placed within the instrument, and ANS spectra were recorded without the addition of ANS. Two microliters of ANS (8 mM) were successively added to yield a 20/40/60/80/100/120 µM ANS in casein solution while awaiting the recording of ANS spectra three minutes in the dark. In the final measurement, 5 µL of ANS was added to reach a final concentration of 150 µM of ANS. The fluorescence intensity was measured using a fluorescence spectrometer (Fluoromax-4 spectrofluorometer, Jobin Yvon, Horiba, Stow, MA, USA) at an excitation and emission wavelength of 390 nm and 470 nm, respectively. The initial slope of the fluorescence intensity against the casein concentration was determined to calculate the protein surface hydrophobicity index (PSHI) by using the following equation [27,28]:

(1) $$PSHI=\frac{\Delta F\;max}{Kd\times \left[prot\right]}$$

With ΔF_max_: maximum number of binding sites where ANS could bind; *Kd*: apparent dissociation constant of an assumed mono-molecular complex (casein-ANS) (µM).

### 2.3. Functional Characterization of Caseins

#### 2.3.1. Functional Characterization of Caseins in Solution

- Emulsifying properties

Emulsions based on demineralized caseins were prepared by mixing 70 g of water, 10 g of protein powder, and 70 g of sunflower oil, respectively, in a kitchen blender. Subsequently, the emulsions were transferred into 100-milliliter glass jars with lids equipped with temperature probes to monitor the thermal treatment applied. The emulsions were sterilized using a static horizontal autoclave with superheated water flow regulated by the MPI Expert program (Steriflow, Paris, France). The viscosity of these emulsions was determined using a Haake™ Viscometer 550 (Thermo Fisher Scientific, Waltham, MA, USA) with the NV geometry before and after heat treatment. Approximately 9 mL of each emulsion was withdrawn from the static module of the NV geometry, and the measurement was conducted in triplicate at a shear rate of 100 s⁻¹ for three minutes at ambient temperature.

- Foaming properties

250 g of 5 wt. % casein solutions were poured into the KitchenAid bowl, and both were introduced on the fridge to reach 5 °C. Then, the solutions were whipped at velocity 10 for 3 min. The overrun rate and foam stability of casein solutions were calculated as described below. Before whipping, the casein solution was transferred into plastic cups at a fixed volume and weighted. The same volume of resulting foam was also weighed using the same plastic cup. The overrun was calculated using the following equation:

(2) $$Overrun\;rate\left(\%\right)=\frac{weight\;of\;casein\;solution-weight\;of\;casein\;foam}{weight\;of\;casein\;foam}\times 100$$

To determine the stability of the foam, a cup filled to the brim with foam was then tipped into a Buchner-type funnel. The time required to totally destabilize the foam was measured.

#### 2.3.2. Functional Characterization of Caseins in Whipping Cream

- Emulsifying properties

A laser granulometer (Shimadzu Europa GmbH SALD-2300, Duisburg, Germany) with a BC23 dispersion cell was used to determine the distribution of the fat globule size. The refractive index and adsorption index of the dispersed phase were set as 1.45 and 0.1, respectively. 1 µL of whipping cream was introduced into the measurement cell, which was filled with deionized water. To determine the level of fat aggregation, 100 µL of whipping cream stocked at 4 °C was taken and diluted in 1 mL of a 1% (*w*/*v*) SDS solution. Thereafter, 10 µL of the mixture was introduced into the water-filled granulometer tank. All measurements were performed three times at room temperature.

The viscosity of the whipping creams was measured using the same method described in Section 2.3.1, with some modifications. The viscosity was evaluated at 541 s⁻¹ at 5 °C. A thermostatic water bath was used to keep the set temperature at ±1 °C of error.

- Foaming properties

500 g of whipping cream was poured into a Kitchen-Aid bowl. The bowl containing the whipping cream and the beater was cooled to 5 °C before whipping. The creams were whipped for 30 s, followed by three minutes of rest using a Planetary beater (Kitchen Aid, USA). At each interval, the overrun rate and firmness were measured. This discontinuous foaming was conducted until the point of churning. The time at which the overrun and firmness were both at their highest was considered the optimal whipping time (OWT), representing the time necessary during whipping to have optimal yield.

After whipping during the OWT determined, the resulting whipped cream was transferred into plastic cups at a fixed volume and weighed. The same volume of unwhipped cream was also weighed using the same plastic cup. The weight was expressed in grams. The overrun was calculated using the following equation:

(3) $$Overrun\;rate\left(\%\right)=\frac{weight\;of\;unwhipped\;cream-weight\;of\;whipped\;cream}{weight\;of\;whipped\;cream}\times 100$$

The firmness of whipped creams was determined in compression by using a TA-XT2i texturometer (Stable Micro Systems, London, UK) using a 35 mm cylindrical probe. The probe penetrated the sample to a depth of 20 mm at a speed of 2 mm/s, and the force exerted on the probe was automatically recorded by the Exponent software version 6.1.7.0 (2014). Firmness was defined as the maximum force required to achieve a given deformation or distance and is expressed in Newton (N) [23]. These parameters were inputted into the application during the testing phase: trigger value at 10 g, pre-test speed at 1 mm/s, and post-test speed at 5 mm/s.

The foam stability was determined as described in 2.3.1 with some modifications. The serum loss over time was quantified after 24 h at ambiant temperature. A foam was considered stable if no exudate was observed after 24 h.

### 2.4. Statistical Analysis

All experiments were repeated at least three times unless specified otherwise. Values were expressed as means ± standard deviation, and the significant difference was analyzed by Tukey’s Test using XLStat 2014 software version 2014.5.03 at a significance level of *p* < 0.05. Different letters accompany statistically different values, and those that are not statistically different have the same letter. In addition, Pair wise correlation was conducted using Pearson correlation analysis to examine the relationship between the functional characteristics and the physicochemical and structural properties of demineralized caseins. The results are visualized in the form of a heatmap created using Excel.

## 3. Results and Discussion

### 3.1. Effect of Demineralization on Physical Properties of Caseins

**Figure 1:**
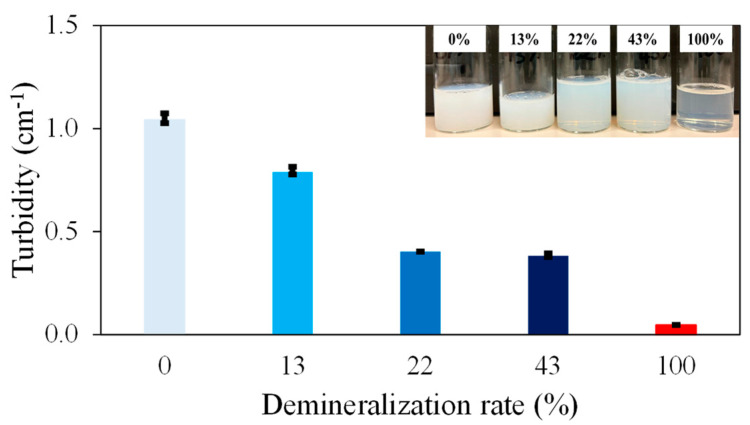
Turbidity measurements in casein solutions measured at 600 nm.

In this study, four caseins with demineralization rates of 0%, 13%, 22%, and 43% were analyzed, using a fully demineralized (100%) commercial caseinate as a reference. First, the effect of demineralization on the casein solution was evaluated by measuring turbidity at 600 nm using a UV-spectrophotometer following centrifugation and filtration (Figure 1).

At 0% demineralization (native caseins), the highest absorbance was observed. When the demineralization level increased from 13% to 43%, the absorbance decreased. The lowest absorbance was recorded for the caseinate (100% demineralization). The variability in turbidity among samples with demineralization rates ranging from 0% to 43% likely reflects differences in casein aggregate size and quantity. The pronounced difference in absorbance between the caseinate and the other samples, as illustrated in Figure 1, suggests significant protein disaggregation at 100% demineralization. These results are consistent with the findings of Ahmadi et al., where turbidity increased as colloidal calcium phosphate levels rose, indicating a decrease in demineralization [15].

**Figure 2:**
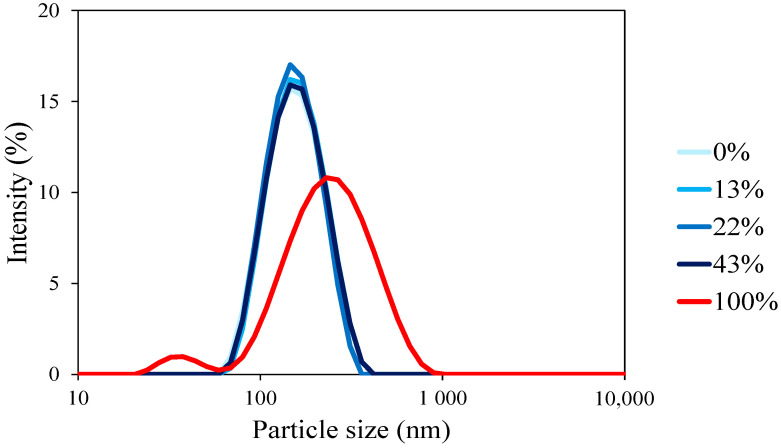
Particle size distribution in nanometers as a function of intensity (%) for different demineralization levels varying from 0 to 100% after centrifugation and filtration.

In order to confirm these conclusions, particle sizes (Z-average diameter) of each casein were determined by using dynamic light scattering (DLS) after centrifugation and filtration to remove possible remaining big aggregates (Figure 2).

Native casein micelles (0% demineralization) displayed a mean diameter of 143 nm, close to the one reported previously for micelle with an average diameter between 150 and 200 nm [4]. For demineralization rates ranging from 13% to 43%, the size of the casein micelles remained consistent with that of native micelles, around 140 nm, and exhibited a monomodal distribution. The findings indicated that there was no evidence of micelle disruption between 0% and 43% of demineralization. This aligns with the observations of Ahmadi et al., who demonstrated that at low demineralization rates (from 100 to 58% of Micellar Calcium Phosphate content), there is no discernible difference in casein size measured by DLS [15]. This technique alone is insufficient to draw definitive conclusions about the effect of demineralization on casein size because DLS cannot distinguish individuals from aggregate micelles [5]. Calculating colloidal calcium phosphate (which was not performed in this study) is necessary to confirm whether partial micelle disruption occurs or if micelle-like aggregates are formed [15].

However, the particle size distribution of the caseinate displayed a bimodal distribution. These findings reveal two primary populations of casein particle sizes: one between 10 and 70 nm, consistent with previous studies representing casein monomers or caseinate aggregates [29,30]. The other peak around 230 nm could be due to the aggregation of casein monomers to form micelle-like aggregates. Lazzaro et al. demonstrated that demineralization can exhibit three distinct populations, each with varying quantities of caseins: micelle-like aggregates (150–200 nm), sodium casein-like aggregates (30–50 nm), and casein monomers [14]. This aggregation could explain the larger particle size observed in the caseinate solution even after centrifugation and filtration. These results were in accordance with the turbidity measurements, with lower absorbance at 100% of demineralization, proving a high level of casein disaggregation. The findings of Li et al. also confirmed this significant disparity in size between the native micelle and sodium caseinate [23].

SANS experiments were then performed at 20 °C to gain insight into the effect of demineralization on the casein at the nanometric scale.

**Figure 3:**
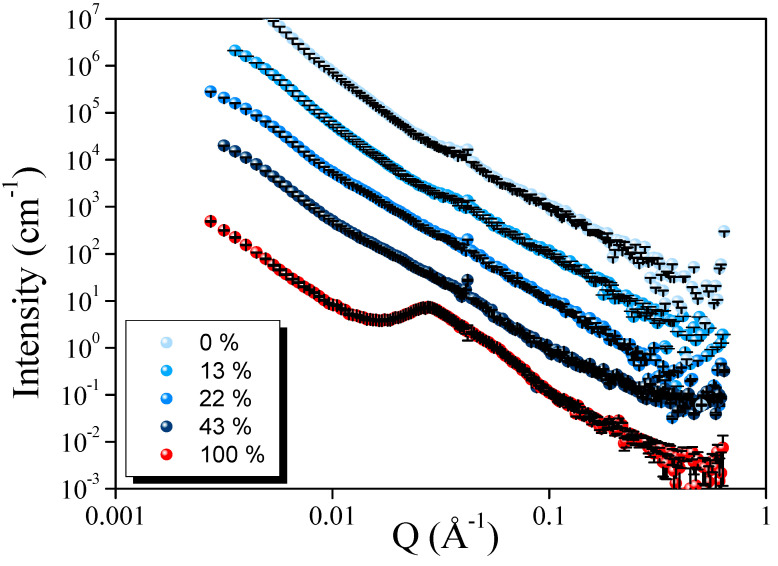
SANS scattering profiles of caseins with different demineralization levels. The scattering curves were shifted vertically for clarity.

Figure 3 displays the scattering profile for all the demineralization rates of the investigated samples varying from 0 % to 100 %. The SANS profiles shifted vertically along the y-axis for clarity. It can be observed that at 100% demineralization, the scattering spectrum exhibited a different scattering pattern in comparison to the other demineralization levels. For caseinate, a scattering maximum peak around 0.027 Å^−1^ was observed. The average packing distance (D) between the caseinate particles could be estimated using D = 2π/Q*max*, resulting in a distance of around 23.3 nm. This value is close to the one already described in the literature for sodium caseinate measured by SAXS measurements [8]. This result is in concordance with DLS measurements, which exhibited a pic around 37 nm for the caseinate. The above-mentioned peak disappeared at a low demineralization level; additionally, all the scattering curves were very similar, from 0 to 43 % of demineralization. The disappearance of the peak observed for these caseins indicates that the particles were no longer present in the aqueous solution. The demineralization rate from 0 to 43% did not induce the formation of casein particles ≤ 100 nm and the scattering curves were similar to the one already described in the literature for casein micelles [31,32]. For all samples, the scattering in the low-Q region (0.002 < Q (Å^−1^) < 0.02) followed a power-law decay with an exponent around 3, indicating that the large-sized particles determined by DLS for all the samples (143–230 nm) possessed either mass or surface fractal-like structures. This result agrees with previous SAXS studies on similar systems [29,30].

Using a multiscale approach, it revealed that demineralization from 0% to 43% resulted in the formation of large casein aggregates predominant even after filtration and centrifugation. This suggests that, despite a decrease in total calcium content, there is a predominance of micelle-like aggregates, suggesting a low impact on micelle structure. However, significant differences were noted in the caseinate (100% demineralization), where the casein was in a monomeric state. The advantage of a low demineralization rate would be to maintain casein in its micelle form, which has been found to enhance the foaming properties [23] but reduce emulsifying properties [14].

### 3.2. Effect of Demineralization on Structural Properties of Caseins

Mid-infrared spectroscopy was used to quantify the secondary structure of the different demineralized casein samples. Milk casein contains a small number of secondary structures [33], but their modifications were proven to influence protein functionalities such as foaming and emulsifying properties [34,35]. MIR enables the characterization and assessment of protein secondary structures through the Amide I, II, and III bands, which are the primary features in the infrared spectrum of proteins [36]. This method also helps to elucidate the interactions (both inter- and intra-molecular) between different regions of a protein. The MIR spectra are depicted in Supporting Information (Figure S1). Calculation of the secondary structure of demineralized caseins in Table 1 exhibited no difference from 0 to 22% demineralization, suggesting that this process has no impact on casein structure up to 22% of demineralization in concordance with Ahmadi et al., who found that demineralization up to 33% result in a retain of protein structure [37].

Any notable discrepancy in random coil content was observed between caseins. The findings of Ahmadi et al. regarding the demineralization rate yielded similar results [15]. Significant differences were observed for side chains and aggregated β-sheets between 0 and 43% demineralization. Only caseinate exhibited notable differences in these secondary structural elements in comparison to the native micelle. Given that side chains can contribute to the structural integrity of proteins through weak interactions, such as ionic or hydrogen bonds [15], these results supported the DLS and SANS measurements, which indicated that any significant changes in micelle structure occurred when the demineralization ranged from 0 to 43%. An increase in β-turns, which can be linked to protein unfolding [37], was observed with increasing demineralization, particularly at 43% and 100% demineralization. These results indicated that contrary to expectations based on side-chain quantification, casein structure undergoes modification when demineralized at 43%. Additionally, Ahmadi et al. observed an increase in β-turns concomitant with a decrease in CCP content (demineralization rate increase) [15]. At 43% and 100% demineralization, there was a reduction in the percentage of β-sheets, accompanied by an increase in the proportion of β-turns and α-helix. These results are partially concordant with the findings of Ahmadi et al., who reported a reduction in CCP attributed to an increase in demineralization, notably affecting β-turns and β-sheets [15]. In contrast with our findings, the authors observed an increase in β-sheets while casein concentrations in CCP significantly decreased. It should be noted that the protocol used to prepare the demineralized protein differed from that employed by the researchers. Instead of alkali, dialysis was used as the neutralizing agent for pH in the present study. It is plausible that the methodology employed may influence the structural characteristics of caseins in varying ways. The reduction in β-sheets observed with increased demineralization, particularly at 43% and 100%, was consistent with the findings of another study by Ahmadi et al., who demonstrated a correlation between the decline in β-sheet and a reduction in CCP levels and the application of heat treatment [37]. This decrease was found to be correlated with either a significant micelle disaggregation or a reduction in the interaction between k-casein and β-lactoglobulin. The increase in α-helices content was also observed by Ahmadi et al. when demineralization occurred at 43 and 100%, concomitant with a reduction in CCP levels to 42% of the original.

From these observations, caseins demineralized at 43% appeared to be a key point at which the structural components of the proteins underwent substantial changes as a result of changes in the demineralization rate (CCP content), despite the presence of disparate physical states with caseinate. The caseins retain some secondary structural features even at strongly acidic pH values, whereas they lose all residual structures at alkaline conditions [38]. From 0 to 22%, there are no difference between samples in terms of structure; it can be postulated that if the emulsifying or foaming properties are correlated to their secondary structures, these caseins will have similar surface-active properties to native casein (good foam stabilizer). Thus, caseins demineralized at 43% and 100% will possess analogous functional properties (good emulsion stabilizer).

The protein surface hydrophobicity serves as an indicator of alterations in protein conformation that affect their functional properties [30]. The evaluation of protein conformation in a particular environment may be conducted utilizing intrinsic (tryptophane) or extrinsic (ANS) fluorophores. The present study investigated the impact of demineralization rate on the internal structure of casein micelles. The surface hydrophobicity of demineralized caseins was quantified by calculating the relative hydrophobicity of amino acid residues exposed on the surface of demineralized caseins that bind ANS. Table 1 indicates PSHI values of demineralized caseins, which decrease when the demineralization rate increases, with the exception of samples demineralized at 43%. At 0% demineralization, the PSHI was the highest for the native micelle. This value decreased at 13% and 22% demineralization. An increase of the PSHI was observed for the casein demineralized at 43%, and the lowest value was obtained for the caseinate. This decrease in protein surface hydrophobicity upon demineralization can be explained by different hypotheses:

(i) The process of demineralization induces the removal of tightly bound calcium, which contributes to stabilizing the protein structure. This destabilization can lead to partial unfolding of the protein, which often exposes hydrophilic (water-attracting) residues that were previously buried in the core, reducing the surface hydrophobicity [39].
(ii) An explanation may also arise from changes in the exposition of hydrophobic segments of the casein micelles. When a protein is partly disorganized, hydrophobic segments can be differently exposed, causing hydrophobic interactions that facilitate protein aggregation, reducing the surface hydrophobicity [40]. Conversely, the secondary structure of demineralized caseins showed only a significant difference at 43 and 100% of demineralization, and the tertiary structure was affected proportionally to the demineralization rate.

These findings suggested that the demineralization rate affected caseins between 43 and 100%, but significant conformation changes were noted when the demineralization rate increased.

### 3.3. Effect of Demineralization on Emulsifying and Foaming Properties of Caseins

Blend fat-based whipping creams were produced using caseins with different demineralization rates ranging from 0 to 100% at the pilot scale. A two-month conservation period was applied to mimic a shelf life following up. Each whipping cream was subjected to an emulsifying and foaming properties characterization. The same or equivalent characterization was performed on caseins in a solution to verify if the casein properties were the same depending on the matrix.

**Figure 4:**
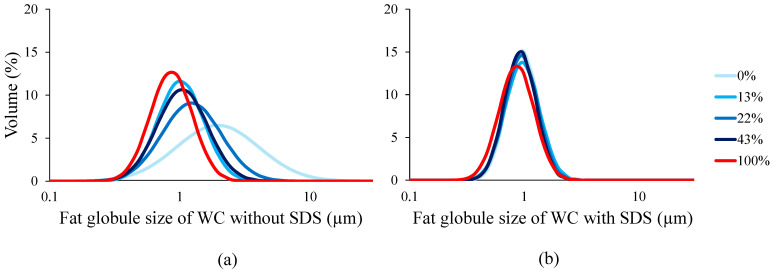
Fat globule size (µm) of whipping creams produced with demineralized caseins (0 to 100%): (**a**) without SDS and (**b**) with SDS.

The emulsifying properties of whipping creams were characterized by measuring the fat globule size distribution and viscosity represented in Figure 4. Emulsion destabilization was positively related to fat globule size and negatively related to viscosity according to Stokes’ law. The results of fat size measurements without the presence of SDS showed the lowest average fat size for caseinate (0.80 µm). This value increases at a low demineralization rate, reaching an average of 1.00 µm at 43%, around 1.12 µm at 22 and 0.95 µm at 13% of demineralization. The whipping cream with native casein exhibits the highest fat globule size (1.81 µm), which is twice that of the other samples. This tendency showed an increase in fat globule size at a low demineralization rate, which suggests a reduction of emulsifying properties [14]. These observations were similar to the presence of SDS, the fat globules slightly increased at a low demineralization rate. The whipping cream prepared with caseinate still has the lowest size (0.81 µm). The samples prepared with casein demineralized from 43 to 0% had an average fat globule size of 0.89 ± 0.2 µm. These findings indicate the presence of fat flocculation on whipping cream prepared with native casein micelle while comparing the size with and without SDS [14].

**Figure 5:**
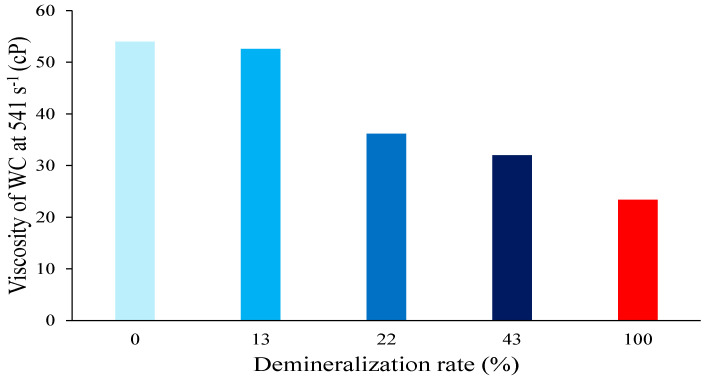
Viscosity of whipping creams produced with different demineralized caseins.

As illustrated in Figure 5, the measurements of viscosity yielded disparate outcomes across the various samples and matrices. The mean viscosity value of the whipping cream samples demonstrated that the lowest viscosity was observed in the caseinate-prepared whipping cream. Subsequently, a low demineralization rate resulted in an observable increase in viscosity, reaching a maximum value for the whipping cream containing native micelle casein. These results are in accordance with those previously reported by Yang et al. [23]. According to Kovacova et al., the observed increase in viscosity can be attributed to the larger fat globule size present in whipping creams containing less demineralized caseins, as previously measured [41]. Given that a high viscosity of whipping cream can also impede the incorporation of air during whipping, a low demineralization rate appears to be disadvantageous for the emulsion. However, whipping cream emulsion must also undergo destabilization (in particular, partial coalescence) in order to form a stable foam. The presence of caseinate, as previously observed, has been demonstrated to enhance the resistance of whipping creams to coalescence which is desirable for whipping creams. Consequently, a low demineralization rate may facilitate the formation of a sufficiently destabilized emulsion, thereby enabling the generation of stable foam.

**Figure 6:**
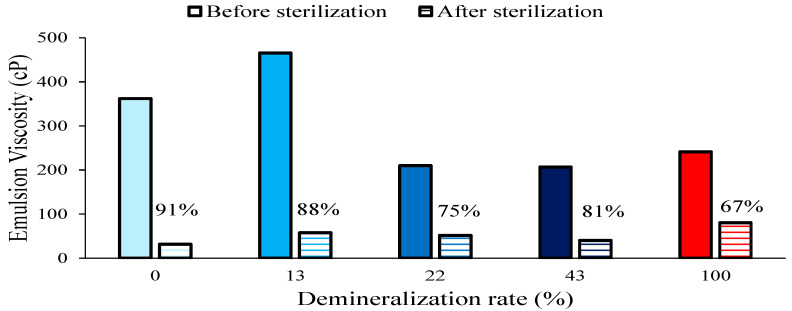
Viscosity of casein emulsions before and after sterilization.

The viscosity of casein emulsions (represented in Figure 6) that have undergone sterilization was found to be greater prior to heat treatment at lower degrees of demineralization, as observed in whipping cream. Following the application of heat treatment, a significant reduction in viscosity was observed. The lowest viscosity was observed for the native micelle, which also exhibited destabilization of the emulsion through partial coagulation at the surface. Viscosity exhibited a relatively consistent profile across the demineralization range of 13% to 43%. At 100% demineralization, the highest stability was observed in terms of viscosity. Except for the native micelle emulsion, which was partially coagulated, all the other samples demonstrated stability after undergoing thermal treatment, and no visible destabilization was observed. The observations of emulsion state and viscosity before and after treatment suggested that the caseinate possesses notable stabilization capacity compared to other samples by allowing a better stabilization of viscosity, which is negatively correlated to emulsification.

The findings indicate that a low demineralization rate may have a detrimental impact on the stability of emulsions in whipped creams. However, it may also result in a level of emulsion destabilization that enhances their foaming properties.

**Figure 7:**
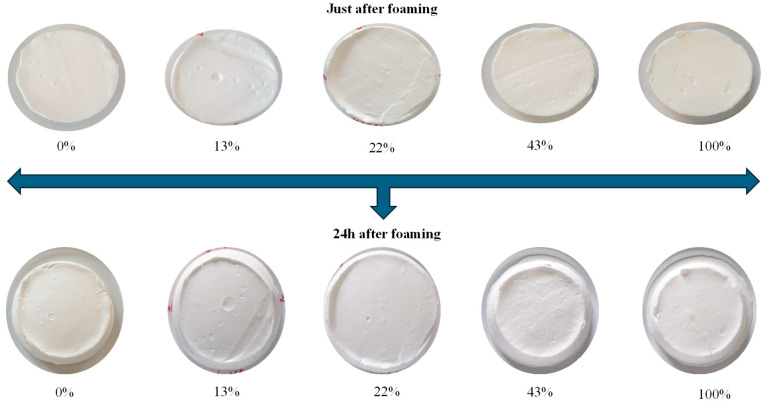
Appearance of foams produced with WC containing demineralized caseins from 0 to 100% just after foaming and 24 h later.

In order to characterize the foaming properties of the whipping creams, the optimum whipping time with a KitchenAid apparatus was determined by employing a discontinuous foaming process prior to whipping. The duration of foam formation was determined based on the point at which the foam reached an optimal state for both overrun and firmness for each sample. As illustrated in Table 2, the highest overrun was observed in the whipping creams containing 43% demineralized casein (316%), while the lowest one was obtained in the native casein micelles (260%). However, the time required to achieve optimal whipping varied among the samples. At 43% demineralization, the shortest time (6 min) was needed to reach the optimum, whereas other samples required more time. No clear trend related to the demineralization rate was observed in this context. Regarding stability, the whipping cream made from less demineralized casein (0% and 13%) was the most stable, with no exudate observed after 24 h (Figure 7). In contrast, the stability of the foams decreased with higher demineralization rates, as seen with caseins demineralized at 22%, 43%, and 100%. These observations align with the measured characteristics of casein foaming in solutions. The findings indicate a significant correlation between foaming properties and the demineralization rate of caseins, independently of the matrix in which the casein is used. These findings corroborate with previous studies [9,23].

### 3.4. Correlation Between Structure, Function, and Application of Whipping Creams

A Pearson correlation with a 95% confidence interval was employed to examine the relationships between structure, function, and application, as illustrated in Table 3. The data are presented in a color-gradient table, ranging from green (positive correlations) to red (negative correlations), with darker shades representing stronger relationships. The analysis of proteins in solutions revealed that a high demineralization rate had a significant impact on casein secondary and tertiary structures. Positive correlations were observed for α-helices (0.76), aggregated β-sheets (0.88), and β-turns (0.94), while negative correlations were found for side chains (−0.99) and hydrophobic sites (−0.83). These structural modifications can impact the emulsifying and foaming properties of demineralized caseins. When focusing on foaming properties, β-sheets, and α-helices can be identified as a key structural element that impacted the foaming characteristics. β-sheets exhibited a negative correlation with both casein foam (−0.84) and whipped cream (−0.95) foaming capacity and a positive correlation with foaming stability of casein solution foam (0.78) and whipping cream (0.76). Conversely, α-helices exhibited a positive correlation with both casein foam (0.71) and whipped cream (0.97) foaming capacity and a negative correlation with foaming stability of casein solution foam (−0.9) and whipping cream (−0.97). However, for foam stabilization, additional secondary structures and surface hydrophobicity were involved. These included a positive correlation with PSHI, β-turns, and side chains. It should be noted that these secondary structures underwent significant modifications at 100% demineralization and also exhibited some impact at 43%. The correlation between β-sheets and foam stability was positive, while the correlation with overrun was negative, and the inverse tendency was observed for the α-helices. This suggests that an increase in β-sheets or a decrease in α-helices would improve stability but impair foaming capacity. The results obtained from the analysis of the secondary structures of the demineralized samples indicate a demineralization rate higher than 13%, which has a positive impact on the overrun but a negative impact on the stability. Side chains demonstrated a notable decline at 100% demineralization relative to the native micelle. A comparable trend was evident for α-helices, which exhibited a marked increase from 43% demineralization. Given that foam stability declines with an increase in the demineralization rate, these findings indicate that the foaming properties are more influenced by their surface hydrophobicity. The correlation between surface hydrophobicity and foam stability was positive, indicating that increased hydrophobicity can enhance foaming stability. This finding aligns with the observations of Othmeni et al. [42]. The rheological properties of the protein at the interface may also influence its foaming properties [43]. Further investigation is required to gain a comprehensive understanding of their impact on foam formation and stability. Overall, the results indicate that at a low demineralization rate, foam stability is enhanced, with a slight effect on the reduction of foaming capacity. The most stable foams were achieved with demineralization levels between 0% and 13%, and minimal impact was observed on the secondary structure of caseins.

With regard to the emulsifying properties of demineralized caseins, the dimensions of fat globules with and without SDS, as well as the viscosity of whipping creams, exhibited a markedly negative correlation with α-helices, β-turns, and positive correlation with surface hydrophobicity and side chains. A contrasting trend was observed in the viscosity of casein emulsions, which exhibited varying correlations with secondary and hydrophobic characteristics of caseins depending on the thermal treatment stage (before and after). In whipping creams, the size of fat globules is negatively associated with the emulsion stability [14]. The differences in emulsifying capacity can be attributed to the surface activity and/or the size of the emulsifying agent. It can be reasonably deduced that the higher the surface activity and/or the higher the emulsifier size is, the lower the emulsifying capacity. At a high degree of demineralization, a decrease in the size of the side chains is observed. Given that it is a constituent of the casein structure, the observations confirm that low demineralization is disadvantageous for emulsifying capacity. The viscosity, which plays a role in stabilizing the colloidal phase against drainage, was found to be greatly correlated with the PSHI. However, depending on the matrix (in solution or whipping after heat treatment), the interfacial behavior differs. This confirms the importance of studying the interfacial properties of the different matrices to gain insight into the internal processes.

Based on the different results obtained from the correlation between the structural characteristics of demineralized caseins and their surface-active properties, surface hydrophobicity seems to be the key structural element affecting both emulsifying and foaming stabilities of caseins. Knowing that emulsification and foaming involve antagonistic mechanisms, an intermediate demineralization rate between 0 and 100% should ameliorate the foaming stability of caseinate with low impact in the emulsion stabilization. The demineralization at 13% seems to be the optimal point based on the different results: good foaming stability, stable emulsion after two months, and low-fat flocculation. The foaming properties exhibited the greatest efficacy at 0% demineralization. However, the emulsion demonstrated instability following heat treatment, and the formation of fat aggregates was observed on WC.

## 4. Conclusions

This study demonstrated that the process of demineralization significantly impacts the secondary and tertiary structures of caseins, influencing their functional properties. The results revealed that demineralization levels above 43% notably affect the size, secondary structures (particularly β-sheets, β-turns, and α-helices), and surface hydrophobicity of caseins. These structural changes enhance emulsifying properties but diminish foam stability. Aggregates were observed across all demineralization levels (13% to 43%) using DLS and SANS experiments. Caseins with low demineralization (notably at 13%) were found to be optimal for applications requiring foam stability, with minimal effects on foam overrun and emulsion properties. WCs prepared with caseins demineralized at 13% provided stable foams while preserving key functionalities such as overrun and emulsification. Conversely, higher demineralization levels (43% to 100%) altered physicochemical properties and reduced effectiveness in foam stabilization, although the ability to form foams remained relatively unaffected. These findings highlight that low demineralization levels (13%) offer a sustainable and functional alternative to caseinates in food applications like whipping creams, delivering a balance between foam stability, overrun, and emulsion stability.

Future research studies could explore optimizing demineralization processes to fine-tune protein functionalities for various food applications. Further investigation into alternative methods of restoring pH, such as the alkali treatment used in this study, could yield more efficient or environmentally friendly techniques. Additionally, studying the impact of demineralization on caseins in more complex food systems beyond whipping creams could broaden the scope of their applications. It would also be valuable to explore the interactions between demineralized caseins and other food components, such as fats or sugars, to better understand how these structural changes influence their performance in real-world formulations. In the future, it would be important to study the impact of low demineralization of caseins not only on their functional properties but also on key organoleptic attributes, such as texture, taste, and appearance. Lastly, innovations in foaming technologies and techniques could further enhance the functionality of low-demineralized caseins, especially in industrial settings requiring high-stability foams.

## Figures and Tables

**Table 1:** Secondary structure and surface hydrophobicity of demineralized caseins from 0 to 100%.

Band Frequency (cm^−1^)	1614–1600	1637–1615	1645–1638	1664–1646	1681–1665	1700–1682	PSHI
Demineralization Rate (%)	Side Chain	β-Sheets	Random Coil	α-Helix	β-Turns	Aggregated β-Sheets	
0	13.6% ^c^	28.6% ^a^	9.1% ^a^	20.9% ^a^	16.9% ^a^	10.9% ^a^	50,000
13	13.4% ^c^	28.2% ^a^	8.8% ^a^	20.7% ^a^	17.2% ^a^	11.7% ^a,b^	33,333
22	13.1% ^b,c^	28.3% ^a^	9.2% ^a^	21.6% ^a,b^	17.2% ^a^	10.7% ^a,b^	25,000
43	12.6% ^b,c^	24.9% ^b^	9.2% ^a^	23.2% ^b^	19.0% ^b^	11.1% ^a,b^	33,333
100	11.8% ^b^	22.7% ^b^	8.9% ^a^	22.8% ^b^	19.8% ^b^	14.1% ^b^	14,286

All data are presented as means ± standard deviation (n = 3). Mean values with different letters in the same column are statistically different (*p* > 0.05) according to the Tukey test.

**Table 2:** Foaming characteristics of foam formed from casein solutions and whipping creams. The small letters a-b indicate groups of statistical differences. Values with similar letter have none significant differences between them.

	Casein Solutions			Whipped Creams		
Demineralization Rate	Overrun Rate (%)	Time of Total Destabilization (min)	Whipping Time Optimum (s)	Overrun Rate (%)	Firmness (N)	Quantity of Liquid Exudate (mL)
0%	794 ^d^	100	480	260 ^d^	1.5	0
13%	900 ^b,c^	68	390	269 ^c^	0.9	0.2
22%	932 ^b,c^	55	510	277 ^c^	0.8	8
43%	1047 ^a^	23	360	317 ^a^	1.2	14
100%	895 ^c^	20	570	301 ^b^	1.0	15

**Table 3:** Table of Pearson correlation indexes of demineralized caseins from 0 to 100% using structural elements to understand the correlation between structure-function and application. The data are presented in a color-gradient table, ranging from green (positive correlations) to red (negative correlations), with darker shades representing stronger relationships.

	Demineralization Rate	α-Helix	β-Sheets	β-Turns	Side Chain	Random Coil	Aggregated β-Sheets	PSHI	Casein Foam Overrun	Casein Foam Stability	Overrun WC	Firmness WC	Foam Destabilization WC	Fat Size of WC Without SDS	Fat Size of WC with SDS	Viscosity of WC	Viscosity of Casein Emulsion Before Sterilization	Viscosity of Casein Emulsion After Sterilization
Demineralization rate	1																	
α-helix	0.76	1																
β-sheets	−0.49	−0.88	1															
β-turns	0.94	0.90	−0.75	1														
side chain	−0.99	−0.85	0.59	−0.97	1													
random coil	−0.24	0.32	−0.29	−0.10	0.11	1												
aggregated β-sheets	0.88	0.40	−0.16	0.75	−0.80	−0.63	1											
PSHI	−0.83	−0.54	0.25	−0.67	0.82	0.25	−0.69	1										
Casein Foam Overrun	0.29	0.71	−0.84	0.48	−0.41	0.35	−0.09	−0.38	1									
Casein Foam Stability	−0.84	−0.90	0.78	−0.90	0.90	−0.03	−0.54	0.80	−0.76	1								
Overrun WC	0.72	0.97	−0.95	0.88	−0.80	0.22	0.37	−0.54	0.82	−0.93	1							
Firmness WC	−0.19	0.06	−0.19	0.01	0.17	0.27	−0.16	0.70	−0.27	0.33	−0.01	1						
Foam destabilization WC	0.84	0.97	−0.76	0.90	−0.91	0.26	0.49	−0.72	0.66	−0.94	0.93	−0.14	1					
Fat size of WC without SDS	−0.68	−0.52	0.47	−0.64	0.69	0.37	−0.56	0.86	−0.64	0.84	−0.64	0.69	−0.62	1				
Fat size of WC with SDS	−0.99	−0.75	0.44	−0.92	0.98	0.19	−0.87	0.79	−0.20	0.78	−0.67	0.12	−0.83	0.58	1			
Viscosity of WC	−0.89	−0.43	0.18	−0.74	0.83	0.60	−0.95	0.87	−0.10	0.67	−0.43	0.46	−0.57	0.77	0.86	1		
Viscosity of casein emulsion before sterilization	−0.52	−0.80	0.55	−0.56	0.61	−0.69	−0.07	0.48	−0.55	0.66	−0.69	0.07	−0.84	0.25	0.54	0.16	1	
Viscosity of casein emulsion after sterilization	0.80	0.29	−0.02	0.60	−0.73	−0.62	0.88	−0.90	0.04	−0.59	0.30	−0.60	0.46	−0.77	−0.77	−0.98	−0.11	1

## Data Availability

The original contributions presented in this study are included in the article/Supplementary Materials. Further inquiries can be directed to the corresponding author.

## Supplementary Materials

The following supporting information can be downloaded at https://www.mdpi.com/article/10.3390/foods13233897/s1, Figure S1. Spectra Mid Infrared of demineralized caseins; Figure S2. Intensity of fluorescence of demineralized caseins from 0 to 100% without ANS; Figure S3. Intensity of fluorescence of demineralized caseins from 0 to 100% with 150 mM of ANS.
