# Supplementary material for: Low Demineralized Caseins to Replace Sodium Caseinate for Application in Whipped Creams
Source: Foods. 2024 Dec 3;13(23):3897. doi: 10.3390/foods13233897 (PMC11640607; doi:10.3390/foods13233897)
Supplement: Supplementary file 1 [file foods-13-03897-s001.zip › foods-3341474-supplementary.pdf]

Supporting Information

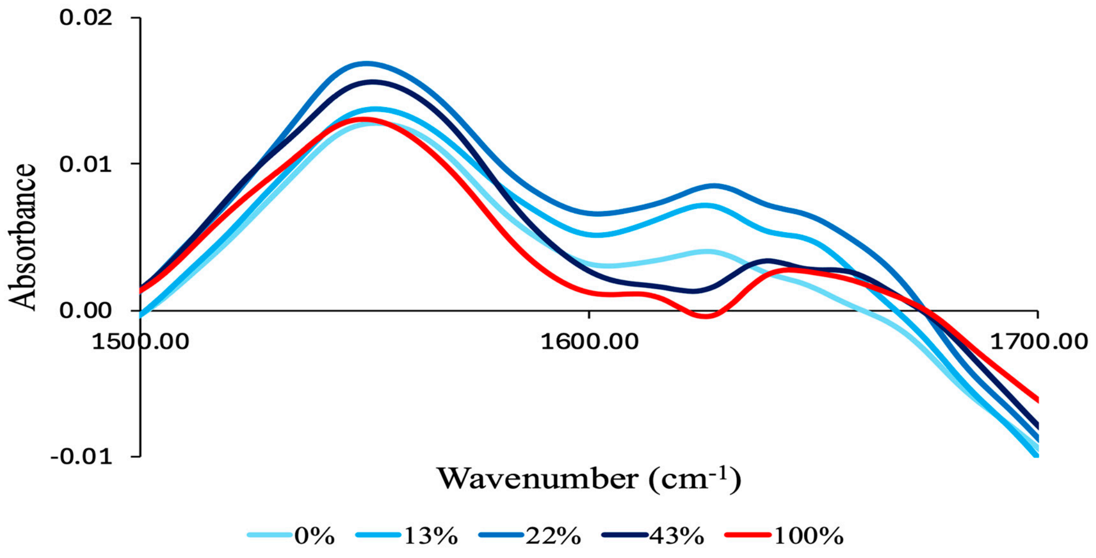

Figure S1. Spectra Mid Infrared of demineralized caseins between 1500 and 1700 cm^{-1}.

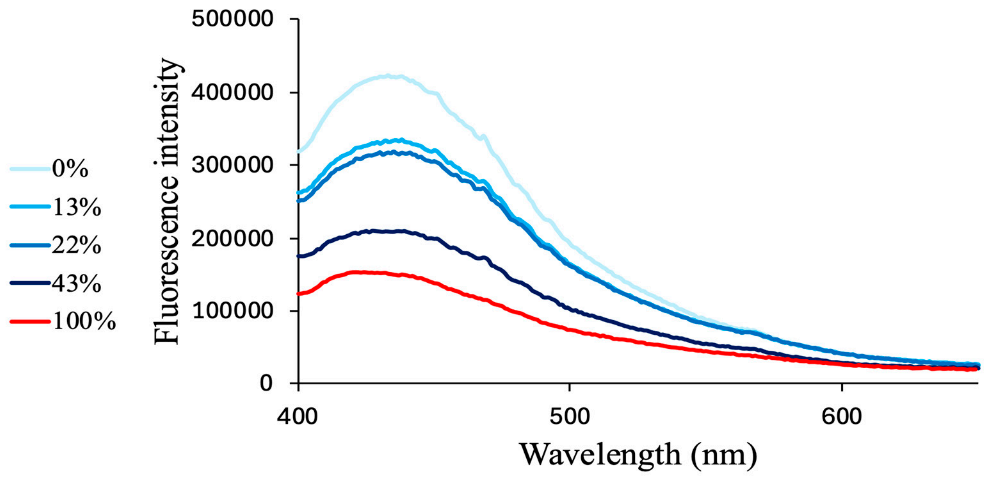

Figure S2. Intensity of fluorescence of demineralized caseins from 0 to 100% without ANS.

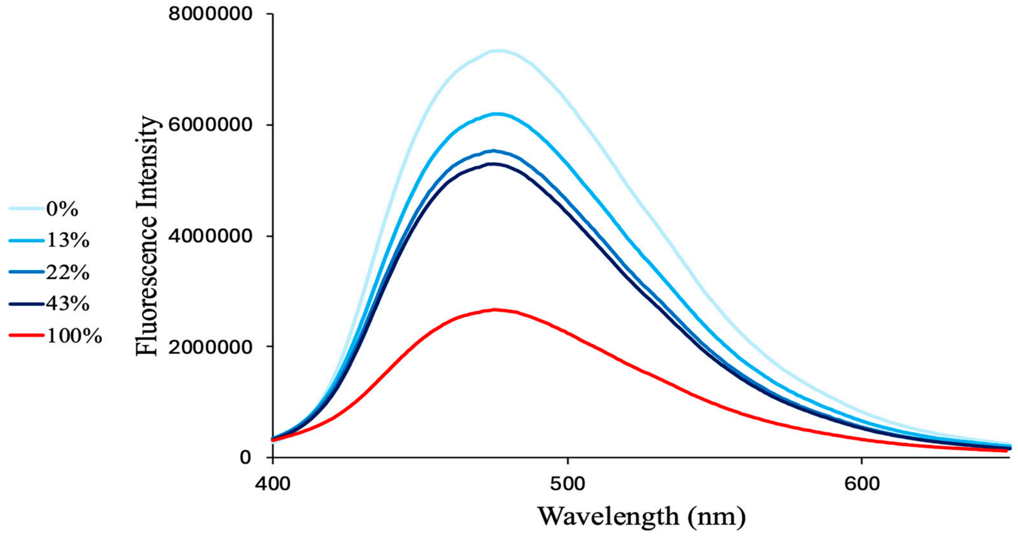

Figure S3. Intensity of fluorescence of demineralized caseins from 0 to 100% with 150 mM of ANS
